# Errata: Low-Dose Anti-PD(L)1 for the Treatment of Solid Malignancies

**DOI:** 10.1200/GO-24-00602

**Published:** 2025-01-09

The article by Souza et al entitled “Low-Dose Anti-PD(L)1 for the Treatment of Solid Malignancies” (*JCO Global Oncology* 10.1200/GO.24.00122) was published online November 21, 2024, with errors.

In the Discussion section, incorrect information was provided for the off-label use of low doses. This paragraph should have read:

To broaden the accessibility of anti-PD(L)1 agents, the off-label use of low doses (and/or at an extended administration interval) is a feasible alternative.^4,23,24^ It has been extensively demonstrated that a linear dose response-toxicity relationship does not exist for anti-PD(L)1 agents.^4,23,24^ Furthermore, pharmacodynamics data support the use of lower doses.^25,26^ For instance, for nivolumab, PD-1 occupancy on circulating T cells seems dose independent, peaking at a mean occupancy of 85% in doses as low as 0.3 mg/kg, **after a single infusion**.^25^ Similarly, for pembrolizumab, ex vivo IL2 stimulation suggests complete peripheral target engagement after doses starting at 1 mg/kg, once **every 2 weeks** with no increments using higher doses.^26^ Finally, pembrolizumab enhanced IL-2 production in cynomolgus monkeys after the lowest doses were tested, indistinguishably from higher doses.^27^

This has been corrected as of January 9, 2025. The authors apologize for the errors.

